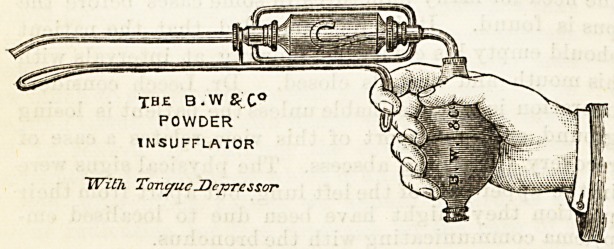# New Appliances and Things Medical

**Published:** 1894-05-05

**Authors:** 


### NEW APPLIANCES AND THINGS MEDICAL.

[All preparations, appliances, novelties, &c., of which a notice is desired, should be sent for the Editor, to care of The Manager, 428, Strand, London, W.C.]

### POWDER INSUFFLATOR.

(Burroughs, Wellcome, and Co., Snow Hill Buildings, London, E.C.)

Messrs. Burroughs and Wellcome have forwarded us a specimen of this new invention. It has many obvious advantages over the older forms. It is a far handier instrument than any of its kind with which we are familiar. As may be seen from the diagram, it is pistol-shaped, and may be managed with one hand, and if used for the throat, can have a tongue depresser fitted to the under surface, thus leaving one hand free for any other purpose that may be necessary. Each instrument is provided with two forms of delivery tube, one straight and the other curved, which are easily and quickly interchangeable. A third tube may also be obtained, having a furcate end, the advantage of which arrangement is that the insufflation can thus be directed in two nebulæ. The operator may, if he desire, have more than one powder cylinder. The advantage of this is obvious. The same cylinder may always be kept for the same powder, and thus time and trouble may be saved in cleaning out the reservoir after use. This instrument is available not only for use in buccal, pharyngeal, laryngeal, and aural practice, but its applicability may be extended to the dusting of surface wounds, sinuses, &c., with antiseptic or astringent powders.

### INSTEP ARCH FOR CURE OF FLAT FOOT.

(Thomas Holland, South Audley Street, W.)

This is a very simple contrivance, and its ingenuity lies in its simplicity. It may be described as an ordinary artificial sock, which is moulded on the inner side to form as it were a valgus pad to support the instep. It is sufficiently rigid to form a very considerable support, and sufficiently comfortable to be objected to by none. It has an advantage over the ordinary valgus pad, because it keeps permanently in position, and over specially-made boots because it is cheaper, and can be worn in the house as well as out of doors. In this respect it offers considerable advantage to nurses. It is certainly worth a trial.

### THE PERFECTED COD-LIVER OIL.

(Allen and Hanbury, Plough Court, Lombard Street.)

The manufacturers of many preparations of cod-liver oil at the present day get over the difficulty of the disagreeable taste and unpleasant after-effects either by disguising the taste with some powerful aromatic flavouring, or by extracting certain chemical principles of the oil, and dishing them up in some palatable form with wine, &c. Messrs. Allen and Hanbury, however, have beaten all previous records by manufacturing a pure cod-liver oil, free from added matter, and hardly more disagreeable than castor oil in flavour. It is a triumph in the art of pharmacy.

### FLEXIBLE SPATULA.

(Henry Steer, Derby.)

A spade is a spade, and a spatula, one would think, must be a spatula; but this particular spatula appears to be different to all others which have preceded it. In the first place it is flexible, and, therefore, can be used for spreading ointments, with the reservation that it must not be used for mercurials since it is silver-plated; and, in the second place, inasmuch as it is silver-plated, it possesses all the advantages of a metal spatula; thirdly, it possesses the merit of costing only 1s.

### HOP GIN.

(J. S. Smith and Co., Phœnix Distillery, London.)

This is a decidedly pleasing preparation of hops and alcohol. The strength of the spirit is about that of a good port wine, and taste is characteristic of the hop. It has very excellent diuretic qualities, and is a perfectly safe soporific to use in cases of sleeplessness. The therapeutic effect of the alcohol is that of a perfectly pure spirit, with no unpleasant after effects.

### SCOTCH WHISKY (Excelsior Brand).

(Margrave Brothers, Llanelly, South Wales.)

Samples of this whisky have been forwarded for our inspection and analysis. We find this whisky perfectly pure, well matured, and of pleasant flavour. Some persons might prefer a whisky of greater alcoholic strength, but to others this mildness will be considered no disadvantage.

## Figures and Tables

**Figure f1:**